# Prevalence and Characterization of Integrons in Multidrug Resistant *Acinetobacter baumannii* in Eastern China: A Multiple-Hospital Study

**DOI:** 10.3390/ijerph120810093

**Published:** 2015-08-21

**Authors:** Jing Chen, Hong Li, Jinsong Yang, Rong Zhan, Aiping Chen, Yansheng Yan

**Affiliations:** 1 Fujian Medical University Union Hospital, No. 29 Xinquan Road, Fuzhou 350001, China; E-Mails: jingchen389@126.com (J.C.); deanzhanrong@aliyun.com (R.Z.); 2 School of Public Health, Fujian Medical University, No.1 Xueyuan Road, Shangjie Town, Minhou County, Fuzhou 350108, China; 3 Department of Pharmaceuticals, Fujian Health College, No. 366 Guankou Road, Jingxi Town, Minhou County, Fuzhou 350101, China; E-Mail: kinglh2002@126.com; 4 Fujian Center for Disease Control and Prevention, 76 Jintai Road, Fuzhou 350001, China; E-Mails: yangjinsong2001@sina.com (J.Y.); cap2006@live.cn (A.C.)

**Keywords:** *Acinetobacter baumannii*, antimicrobial resistance, multidrug resistance, integron, eastern China

## Abstract

*Objective*: The aim of this multiple-hospital study was to investigate the prevalence of integrons in multidrug-resistant *Acinetobacter baumannii* (MDRAB) in Eastern China, and characterize the integron-integrase genes, so as to provide evidence for the management and appropriate antibiotic use of MDRAB infections. *Methods*: A total of 425 clinical isolates of *A. baumannii* were collected from 16 tertiary hospitals in 11 cities of four provinces (Fujian, Jiangsu, Zhejiang and Shandong) from January 2009 to June 2012. The susceptibility of *A. baumannii* isolates to ampicillin/sulbactam, piperacillin/tazobactam, ceftazidime, ceftriaxone, cefepime, aztreonam, meropenem, amikacin, gentamicin, tobramycin, ciprofloxacin, levofloxacin, sulfamethoxazole/trimenthoprim, minocycline and imipenem was tested, and integrons and their gene cassettes were characterized in these isolates using PCR assay. In addition, integron-positive *A. baumannii* isolates were genotyped using pulsed-field gel electrophoresis (PFGE) assay, and *intI1* gene cassette was sequenced. *Results*: *intI1* gene was carried in 69.6% of total *A. baumannii* isolates, while *intI2* and *intI3* genes were not detected. The prevalence of resistance to ampicillin/sulbactam, piperacillin/tazobactam, ceftazidime, ceftriaxone, cefepime, aztreonam, imipenem, meropenem, amikacin, gentamicin, tobramycin, ciprofloxacin, levofloxacin and sulfamethoxazole/trimenthoprim was significantly higher in integron-positive *A. baumannii* isolates than in negative isolates (all *p* values <0.05), while no significant difference was observed in the prevalence of minocycline resistance (*p* > 0.05). PFGE assay revealed 27 PFGE genotypes and 4 predominant genotypes, P1, P4, P7 and P19. The PFGE genotype P1 contained 13 extensive-drug resistant and 89 non-extensive-drug resistant *A. baumannii* isolates, while the genotype P4 contained 34 extensive-drug resistant and 67 non-extensive-drug resistant isolates, appearing a significant antimicrobial resistance pattern (both *p* values <0.05). Sequencing analysis revealed two gene cassette assays of *aacA4-catB8-aadA1* and *dfrXII-orfF-aadA2* in MDRAB isolates. *Conclusions*: The results of this study demonstrate a high prevalence of class 1 integrons in MDRAB in Eastern China, and a greater prevalence of antimicrobial resistance in *intI1* gene-positive MDRAB isolates than in negative isolates. Four predominant PFGE genotypes are identified in *intI1* gene-positive MDRAB isolates, in which P4 is an epidemic PFGE genotype in Fujian Province, and it has a high proportion of extensive drug resistant *A. baumannii*. The gene cassette *dfrXII-orfF-aadA2* is reported, for the first time, in *A. baumannii* strains isolated from Fujian Province, Eastern China.

## 1. Introduction

As a common opportunistic bacterial pathogen of hospital-acquired infections, *Acinetobacter baumannii* has a high ability to acquire resistance to multiple antibiotics and clonal spread [1,2,3]. Multiple-, extensive- or extreme-drug resistant *A. baumannii* is reported worldwide, and the outbreaks of infection with drug resistant strains have become a serious threat to global public health [4,5,6,7]. Integron is a specific integration and exercise system that allows the capture of a single or multiple exogenous gene cassettes, which is of a great significance in the emergence and spread of multidrug resistant *A. baumannii* (MDRAB) [8,9,10]. A regional variation has been detected in the type and gene cassette structure of integron in MDRAB strains [11,12,13]. Therefore, understanding of the epidemiological features and spread pattern may provide new insights into the management of *A. baumannii* infections and antibiotic resistance [14,15,16]. The major purposes of this multiple-hospital study were to investigate the prevalence of integrons in MDRAB strains isolated from 16 tertiary hospitals in Eastern China, and characterize the integron-integrase genes, in order to provide evidence for the management and appropriate antibiotic use of MDRAB infections.

## 2. Materials and Methods

### 2.1. A. baumannii Isolates

A total of 514 clinical bacterial isolates, identified as MDRAB using VITEK 2 (bioMérieux; Marcy l’Etoile, France), were collected from 16 tertiary hospitals in 11 cities of four Chinese provinces (Fujian, Jiangsu, Zhejiang and Shandong) during the period from January 2009 through June 2012. Then, a one-tube multiplex PCR assay was performed for rapid identification of *A. baumannii* using the method described previously [17]. Accordingly, 425 isolates were identified as *A. baumannii* (Table 1), while the other 89 isolates belonged to other species of *Acinetobacter*.

**Table 1:** Number of *A. baumannii* strains isolated from 16 hospitals in eastern China.

Province	City	Hospital Code	Number of *A. baumannii* Strains Isolated
Fujian	Fuzhou	A	254
		B	2
		C	20
		D	15
	Xiamen	E	25
		F	1
		G	3
	Quanzhou	H	14
	Longyan	I	5
	Nanping	J	56
Jiangsu	Nanjing	K	3
Zhejiang	Haining	L	3
	Hangzhou	M	17
	Taizhou	N	2
	Wenzhou	O	2
Shandong	Yantai	P	3
Total			425

### 2.2. Antimicrobial Susceptibility Test

The susceptibility of *A. baumannii* to ampicillin/sulbactam, piperacillin/tazobactam, ceftazidime, ceftriaxone, cefepime, aztreonam, meropenem, amikacin, gentamicin, tobramycin, ciprofloxacin, levofloxacin, sulfamethoxazole/trimenthoprim, minocycline (National Institutes for Food and Drug Control, Beijing, China) and imipenem (Merck Sharp & Dohme Pharmaceutical Co., Ltd., Shanghai, China) was tested using the Clinical Laboratory Standard Institute (CLSI) recommended agar dilution method [18], and was evaluated using the CLSI interpretive criteria [19]. *Escherichia coli* ATCC25922 and *Pseudomonas aeruginosa* ATCC27853 (Shanghai Fuxiang Biotech Co., Ltd., Shanghai, China) served as quality control bacterial isolates.

### 2.3. Characterization of Integrons and Their Gene Cassettes

The bacterial colonies of *A. baumannii* were prepared into bacterial suspensions, boiled at 100 °C for 10 min in a DK-8D thermostatic water bath (Shanghai Boheng Scientific Instruments Co., Ltd., Shanghai, China), and then centrifuged at 8000 r/min for 10 min at room temperature. The supernatant was collected and used as a DNA template for the subsequent PCR assay.

Characterization of class 1 (*intI1*), 2 (*intI2*) and 3 (*intI3*) integron-integrase genes and *intI1* gene cassette (*intI CS*) was done using a PCR assay in a 50 μL system containing 5 μL of 10 × PCR buffer, 1 μL of each forward and reverse primer reported previously (Table 2) [20], 4 μL of dNTPs (Takara, Dalian, China), 0.3 μL of Taq DNA polymerase (Takara), 5 μL of DNA template, and 33.7 μL of ddH_2_O. The conditions for PCR amplification were: initial denaturation at 95 °C for 5 min, followed by 30 cycles of denaturation at 95 °C for 30 s, primer annealing at 55 °C for *intI1* and *intI2*, 50 °C for *intI3* and 46 °C for *intI CS* for 30 s, and extension at 72 °C for 30 (for *intI1* and *intI2*) or 60 s (for *intI3* and *intI CS*), and final extension at 72 °C for 7 min. The PCR amplification products were checked with electrophoresis on a 1.5% agarose gel. In addition, the variable region of integrons was amplified using the specific primers targeting the 5’ and 3’ conserved regions, purified, and sequenced by the Shanghai Biosune Biotechnology Co., Ltd. (Shanghai, China). Then, the type and sequence of the gene cassette were identified through homology analysis using the software BLAST [21,22,23].

**Table 2:** Primer sequences and PCR product size of integrase genes.

Integrase Gene	Primer Sequences	PCR Product Size (bp)	Reference
*intl1*	F: 5’-CAG TGG ACA TAA GCC TGT TC-3’;	160	20
	R: 5’-CCC GAG GCA TAG ACT GTA-3’		
*intl2*	F: 5’-TTG CGA GTA TCC ATA ACC TG-3’;	288	20
	R: 5’-TTA CCT GCA CTG GAT TAA GC-3’		
*intl3*	F: 5’-GCCTCCGGCAGCGACTTTCAG-3’;	1041	20
	R: 5’-ACGGATCTGCCAAACCTGACT-3’		
*intl CS*	F: 5’-GGC ATC CAA GCA GCA AG-3’;	Unknown	20
	R: 5’-AAGCAG ACT TGA CCT GA-3’		

### 2.4. Strain Typing of Integron-Positive A. baumannii Isolates

The integron-positive *A. baumannii* isolates were genotyped using pulsed-field gel electrophoresis (PFGE) [24], while *Salmonella* serotype Braenderup H9812 strain (PulseNet China, Beijing, China) served as a standard quality control isolate. Briefly, genomic DNA was extracted from integron-positive *A. baumannii* isolates, and digested with restriction endonuclease *Apa I* (New England Biolabs, Inc., Beverly, MA, USA). Electrophoresis was performed at 14 °C on a 1% Seakem Gold agarose gel (Lonza, Rockland, ME, USA) in the CHEF Mapper® Pulsed Field Electrophoresis System (Bio-Rad, Hercules, CA, USA) for 19 h under the following conditions: switching time of 5 to 20 s, 120° field angle, and voltage of 6 V/cm. Gel images were captured using the Gel Doc XR^+^ imaging system (Bio-Rad), and all PFGE profiles were processed using the software BioNumerics version 6.0 (Applied Maths, Inc., Austin, TX, USA). Similarities were obtained using the Dice coefficient at a 1.5% tolerance, and a dendrogram was constructed with the unweighted-pair group method using average linkages (UPGMA) clustering method. A mean similarity of <90% indicated various PFGE genotypes, whereas a similarity of 90% or greater was defined as the same PFGE genotype [24].

### 2.5. Ethical Statement

This study was approved by the institutional review board of Fujian Medical University Union Hospital and Fujian Medical University. All experimental procedures performed in this study complied with all laws and regulations in China.

### 2.6. Statistical Analysis

All antimicrobial susceptibility testing results were analyzed using the software WHONET version 5.6, and all statistical analyses were performed with the statistical software SPSS 14.0 (SPSS Inc., Chicago, IL, USA). The differences of the prevalence of antimicrobial resistance were compared with chi-square test. A *P* value <0.05 was considered statistically significant.

## 3. Results

### 3.1. Prevalence of intI1, intI2 and intI3 Genes in A. baumannii

**Figure 1:**
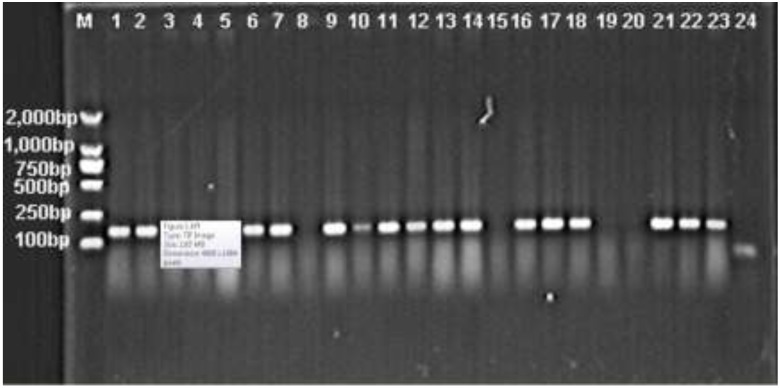
Electrophoresis of the PCR amplification products of *intI1* gene. M, DL 2000 DNA Marker; 1–23, *A. baumannii* isolates; 24, Negative control isolate. Positive bands show the PCR products of *intI1* gene.

Among the 425 clinical isolates of MDRAB, there were 296 isolates (69.6%) positive for *intI1* gene, while *intI2* and *intI3* genes were not detected (Figure 1).

### 3.2. Comparison of the Prevalence of Antimicrobial Resistance between Integron-Positive and -Negative A. baumannii Isolates

A significantly higher prevalence of resistance to ampicillin/sulbactam, piperacillin/tazobactam, ceftazidime, ceftriaxone, cefepime, aztreonam, imipenem, meropenem, amikacin, gentamicin, tobramycin, ciprofloxacin, levofloxacin and sulfamethoxazole/trimenthoprim was detected in integron-positive *A. baumannii* isolates than in integron-negative isolates (all *p* values <0.05); however, no significant difference was observed in the prevalence of minocycline resistance between these two groups isolates (Table 3).

**Table 3:** Comparison of the prevalence of antimicrobial resistance between integron-positive and -negative *Acinetobacter baumannii* isolates.

Antimicrobial	Integron-Positive *A. baumannii* Isolates (*n* = 296)	Integron-Negative *A. baumannii* Isolates (*n* = 129)	*p* Value
Ampicillin/sulbactam	89.9%	62.4%	<0.05
Piperacillin/tazobactam	92.7%	65.2%	<0.05
Ceftazidime	98.5%	75.4%	<0.05
Ceftriaxone	100%	98%	<0.05
Cefepime	94.3%	74.6%	<0.05
Aztreonam	99.6%	93.1%	<0.05
Imipenem	86.6%	65.8%	<0.05
Meropenem	89.9%	57.6%	<0.05
Amikacin	87.8%	42.7%	<0.05
Gentamicin	98.1%	71.7%	<0.05
Tobramycin	93.3%	62.7%	<0.05
Ciprofloxacin	98.7%	77.7%	<0.05
Levofloxacin	86.2%	55.4%	<0.05
Sulfamethoxazole/trimenthoprim	98.3%	85.3%	<0.05
Minocycline	39.3%	28.8%	0.119

### 3.3. PFGE Analysis of intI1 Gene-Positive A. baumannii Isolates

**Figure 2:**
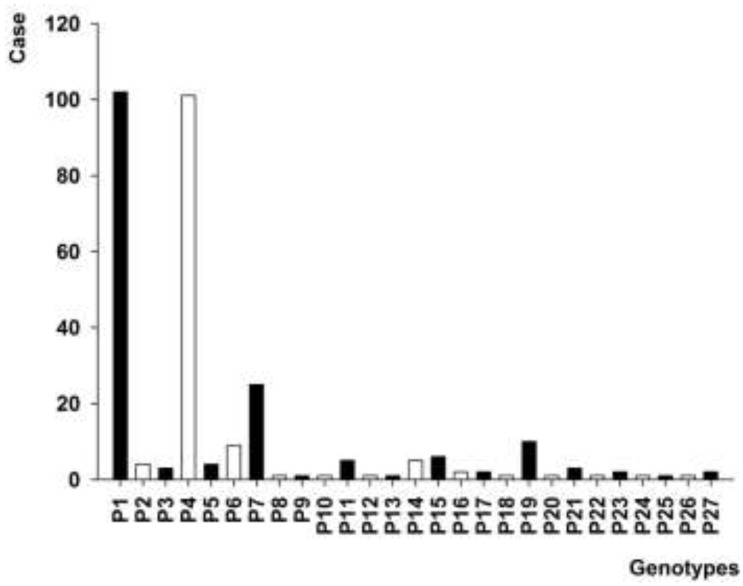
PFGE profile of 296 *A. baumannii* isolates positive for *intl1* gene.

PFGE assay of 296 *intI1* gene-positive *A. baumannii* isolates revealed 27 PFGE genotypes, and there were 4 genotypes containing 10 or more *A. baumannii* isolates, including genotypes P1 (102 isolates), P4 (101 isolates), P7 (25 isolates) and P19 (10 isolates) (Figure 2).

### 3.4. Distribution of intI1 Gene-Positive A. baumannii Isolates

**Figure 3:**
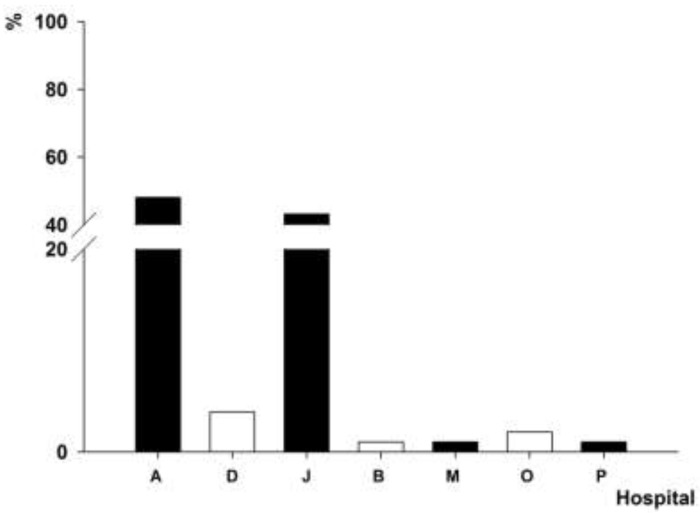
Distribution of *A. baumannii* isolates with PFGE genotype P1.

**Figure 4:**
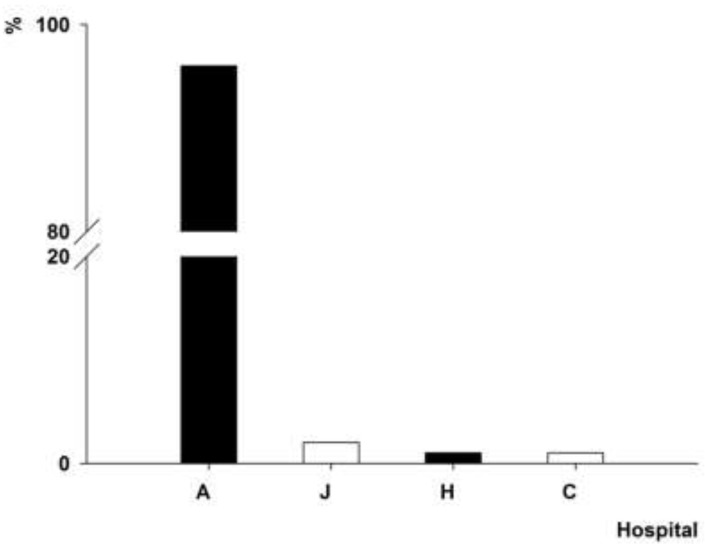
Distribution of *A. baumannii* isolates with PFGE genotype P4.

**Figure 5:**
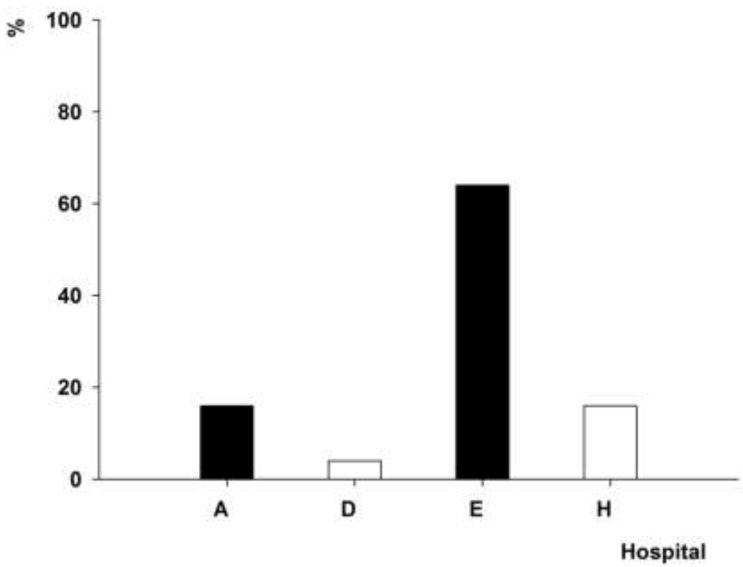
Distribution of *A. baumannii* isolates with PFGE genotype P5.

The *intI1* gene was detected in the clinical strains of *A. baumannii* isolated from 15 hospitals. The PFGE genotype P1, which contained 102 *A. baumannii* isolates, was detected in the *A. baumannii* strains isolated from hospitals A (48%) and J (43.1%) (Figure 3). The PFGE genotype P4, which contained 101 *A. baumannii* isolates, was found in the *A. baumannii* strains isolated from four hospitals in two cities (Figure 4), 96% of which was detected in the *A. baumannii* strains isolated from A hospital. The PFGE genotype P7, which contained 25 *A. baumannii* isolates, was prevalent in the *A. baumannii* strains isolated from four hospitals in three cities (Figure 5), 64% of which was detected in the *A. baumannii* strains isolated from hospital E. The PFGE genotype P9 contained 10 *A. baumannii* strains, which were isolated from hospitals A (seven isolates), I (two isolates) and D (one isolate).

### 3.5. Antimicrobial Resistance Pattern of intI1 Gene-Positive A. baumannii Isolates with PFGE Genotypes P1 and P4

PFGE assay revealed that P1 and P4 were the predominant PFGE genotypes in the 296 *intI1 gene*-positive *A. baumannii* isolates. The PFGE genotype P1 contained 13 extensive-drug resistant and 89 non-extensive-drug resistant *A. baumannii* isolates, and the PFGE genotype P4 contained 34 extensive-drug resistant and 67 non-extensive-drug resistant *A. baumannii* isolates. There were significant differences observed in the antimicrobial resistance pattern (both *p* values <0.05).

### 3.6. Sequencing of int I Gene Cassette

The variable region of integrons was detected in 115 *A. baumannii* isolates positive for *intI1* gene, in which two arrays of cassettes, with 2.3 kb (97 isolates) and 1.8 kb (18 isolates) in lengths, were detected. Sequence alignment revealed that the sequences of these two arrays of gene cassettes had both 99% homology with the sequences of the two cassettes (GenBank accession number: AY557339 and AB154407) within the variable regions of class I integron of *A. baumannii*, which encoded the drug resistance gene cassettes *aacA4-catB8-aadA1* and *dfrXII-orfF-aadA2*.

## 4. Discussion

The long-term extensive use of antimicrobial agents may lead to the emergence of antimicrobial resistance [25]. Elucidation of the mechanism underling the antimicrobial resistance would be of great importance for the prevention and control of the spread of drug-resistant bacteria [26]. Under the selective pressure of antibiotics, MDRAB may emerge due to gene mutations and acquisition of exogenous drug resistance genes [27]. Horizontal transfer of drug resistance genes may occur within and among bacterial species [28], and integron-mediated horizontal transfer of drug resistance genes has been paid more and more attention [29,30]. It is reported that integrons play a vital role in the horizontal transfer of drug resistance genes in Gram-negative bacilli [31].

Integrons are classified according to the sequence of the integrase gene. Currently, class 1 and 2 integrons are the most common types detected in Gram-negative bacteria, and class 1 integron shows the highest prevalence in *A. baumannii* [32]. It has been shown that there are 40%–92% of *A. baumannii* strains carrying class 1 integrons, with a low prevalence of class 2 integrons detected, and class 1 integrons and their gene cassettes are a major contributor to the emergence of MDRAB [11,33,34]. Detection of integrons is therefore considered to serve as an indicator to assess the epidemics of *A. baumannii* [8,9,10]. In the present study, 69.6% of MDRAB isolates were found to carry *intI1* gene, with no *intI2* gene detected, and PFGE assay of the *intI1* gene-positive *A. baumannii* isolates revealed four predominant epidemic clones, including genotypes P1, P4, P7 and P19, which were epidemic in 10 hospitals sampled from seven cities. Since a large number of *A. baumannii* strains were isolated from hospital A during a long period of time, the *intI1* gene-positive *A. baumannii* clinical isolates from hospital A were characterized 14 PFGE genotypes, and a high proportion of P1 and P4 genotypes was detected, notably P4 (96%). In relative to other PFGE genotypes, the clinical *A. baumannii* isolates with P1 genotype were distributed in seven hospitals from five cities. Hospital A is a large tertiary teaching hospital, and critically ill patients throughout Fujian Province are admitted to the hospital, which results in the inter-hospital spread of *intI1* gene-positive MDRAB.

It has been proved that integrons are involved in the development of MDR in *A. baumannii* [12,15,30]. A higher prevalence of antibiotic resistance is detected in integron-positive MDRAB than in negative strains [34,35]. Detection of 48 epidemic clinical strains of *A. baumannii* isolated from 11 hospitals showed a 50% carriage of integrons in the tested bacterial strains, and a greater prevalence of MDR was detected in integron-positive strains than in negative strains, demonstrating the important role of integrons in antibiotic resistance and thereby in the epidemic behavior of *A. baumannii* [36]. Our findings showed a significantly greater prevalence of resistance to ampicillin/sulbactam, piperacillin/tazobactam, ceftazidime, ceftriaxone, cefepime, aztreonam, imipenem, meropenem, amikacin, gentamicin, tobramycin, ciprofloxacin, levofloxacin and sulfamethoxazole/trimenthoprim was detected in integron-positive *A. baumannii* isolates than in negative isolates (all *p* values <0.05), which was in agreement with previous reports [34,35,36]. In addition, we compared the antimicrobial resistance pattern of P1 and P4 clones, two predominant PFGE genotypes in the 296 *intl1 gene*-positive *A. baumannii* isolates, and significant differences were found (both *p* values <0.05). The PFGE genotype P4 contained more extensive-drug resistant *A. baumannii* isolates, and this clone was found to be the predominant epidemic genotype in hospital A. Therefore, the strengthening of the implementation of interventions targeting the management of MDRAB infections and appropriate use of antibiotics are required.

The category and number of integron-carried gene cassettes are strongly associated with the development of MDR in *A. baumannii* [9,15]. The regional variation of epidemic MDRAB clones leads to various epidemic characteristics of their drug resistance gene cassettes. Among the 65 *A. baumannii* isolates collected from four regional hospitals in northern Taiwan in 2009, approximately 72% carriage of *intI1* genes was detected in the *A. baumannii* isolates, which mainly carried three gene cassette arrays of *aacC1-orfX-orfX-orfX’-aadA1a*, *aacA4-catB8-aadA1* and *dfrXII-orfF-aadA2* [37]. Detection of class 1 and class 2 integrons in *A. baumannii* isolates from nine hospitals in Turkey showed 6.4% (18/281) prevalence of class 1 integrons and absence of class 2 integrons in the *A. baumannii* isolates, and the gene cassettes of class 1 integrons *AacC1-AAC(3)I-aadA1*, *AacC1-aadA1*, *AAC(3)-I*, *AAC(3)-I-AAC(3)-I-aadA1*, *TEM-1*, *AAC(3)-I-aadA1-AAC(3)-I-AAC(3)-I*, *AAC(3)-I-AAC(3)-I-AAC(3)-I-aadA1*, *AAC(3)-I-aadA1*, *AAC(3)-I- AAC(3)-I*, *AAC(3)-I-aadA1-AAC(3)-I-aadA1* and *AAC(3)-I-AAC(3)-I-aadA1-AAC(3)-I-aadA1* were detected in 18 isolates [22]. In the United Kingdom, class 1 integrons were found in all of the outbreak isolates of *A. baumannii* but in none of the sporadic isolates, and integrons were recognized as useful markers for the outbreak of epidemic strains of *A. baumannii*; in addition, four integron cassette arrays were reported, including *aacA4*, *aacA4-catB8-aadA1*, *aacC1-orfX-orfX’-aadA1a* and *aacC1-orfX- orfX-orfX’-aadA1a* [11], which have been found in other outbreak strains of *A. baumannii* from Taiwan [37], Italia [38] and some other European countries [39]. In addition, class 1 integrons were detected 52.8% of the *A. baumannii* isolates collected from Nanjing, China, which mainly carried gene cassette arrays of *orf1-aadA1* and *aacA4-catB8-aadA1*. In the current study, the gene cassette arrays of *aacA4-catB8-aadA1* and *dfrXII-orfF-aadA2* were detected. The *aacA4-catB8-aadA1* is a common gene cassette worldwide; however, *dfrXII-orfF-aadA2* is only epidemic in eastern China [40]. This is the first report of the gene cassette *dfrXII-orfF-aadA2* in *A. baumannii* strains isolated from Fujian Province, Eastern China. In these two gene cassettes, *aacA4* encodes aminoglycoside 6’-N-acetyltransferase, which results in the resistance to amikacin, netilmicin, and tobramycin; *aadA1* encodes aminoglycoside nucleotidyltransferase, which induces the resistance to streptomycin and spectinomycin; *catB8* encodes chloramphenicol acetyltransferase, which leads to the resistance to chloromycetin; *orfF* is a gene with unknown function; *dhfrXII* is a dihyrofolate reductase gene, which mediates the resistance to trimethoprim and streptomycin; and *aadA2* is a streptomycin adenylyltransferase gene, which is involved in the resistance to streptomycin and spectinomycin. Therefore, the drug resistance genes carried by these two gene cassettes are involved in the development of resistance to aminoglycosides and sulfonamides. However, no drug resistance genes involved in the resistance to carbapenems were detected in these two gene cassettes, inferring the complex mechanisms of antimicrobial resistance in *A. baumannii*.

## 5. Conclusions

A high prevalence of class 1 integrons is detected in MDRAB in Eastern China, and the prevalence of antimicrobial resistance is greater in *intI1* gene-positive MDRAB isolates than in negative isolates. There are four predominant PFGE genotypes in *intI1* gene-positive MDRAB isolates, in which P4 is an epidemic PFGE genotype in Fujian Province, and it has a high proportion of extensive drug resistant *A. baumannii*. We report the gene cassette dfrXII-orfF-aadA2, for the first time, in *A. baumannii* strains isolated from Fujian Province, Eastern China.

## References

[1] Zhang H.Z., Zhang J.S., Qiao L. (2013). The *Acinetobacter baumannii* group: A systemic review. World J. Emerg. Med..

[2] Howard A., O’Donoghue M., Feeney A., Sleator R.D. (2012). *Acinetobacter baumannii*: An emerging opportunistic pathogen. Virulence.

[3] Peleg A.Y., Seifert H., Paterson D.L. (2008). *Acinetobacter baumannii*: Emergence of a successful pathogen. Clin. Microbiol. Rev..

[4] Antunes L.C., Visca P., Towner K.J. (2014). *Acinetobacter baumannii*: Evolution of a global pathogen. Pathog. Dis..

[5] Durante-Mangoni E., Zarrilli R. (2011). Global spread of drug-resistant *Acinetobacter baumannii*: Molecular epidemiology and management of antimicrobial resistance. Future Microbiol..

[6] Giamarellou H., Antoniadou A., Kanellakopoulou K. (2008). *Acinetobacter baumannii*: A universal threat to public health?. Int. J. Antimicrob. Agents.

[7] Gootz T.D., Marra A. (2008). *Acinetobacter baumannii*: An emerging multidrug-resistant threat. Expert. Rev. Anti Infect. Ther..

[8] Chang-Tai Z., Yang L., Zhong-Yi H., Chang-Song Z., Yin-Ze K., Yong-Ping L., Chun-Lei D. (2011). High frequency of integrons related to drug-resistance in clinical isolates of *Acinetobacter baumannii*. Indian J. Med. Microbiol..

[9] Lee Y.T., Huang L.Y., Chen T.L., Siu L.K., Fung C.P., Cho W.L., Yu K.W., Liu C.Y. (2009). Gene cassette arrays, antibiotic susceptibilities, and clinical characteristics of *Acinetobacter baumannii* bacteremic strains harboring class 1 integrons. J. Microbiol. Immunol. Infect..

[10] Ruiz J., Navia M.M., Casals C., Sierra J.M., Jiménez De Anta M.T., Vila J. (2003). Integron-mediated antibiotic multiresistance in *Acinetobacter baumannii* clinical isolates from Spain. Clin. Microbiol. Infect..

[11] Turton J.F., Kaufmann M.E., Glover J., Coelho J.M., Warner M., Pike R., Pitt T.L. (2005). Detection and typing of integrons in epidemic strains of *Acinetobacter baumannii* found in the United Kingdom. J. Clin. Microbiol..

[12] Wu T.L., Ma L., Chang J.C., Su L.H., Chu C., Leu H.S., Siu L.K. (2004). Variable resistance patterns of integron-associated multidrug-resistant *Acinetobacter baumannii* isolates in a surgical intensive care unit. Microb. Drug. Resist..

[13] Oh J.Y., Kim K.S., Jeong Y.W., Cho J.W., Park J.C., Lee J.C. (2002). Epidemiological typing and prevalence of integrons in multiresistant *Acinetobacter* strains. Acta. Pathol. Microbiol. Immunol. Scand..

[14] Labbate M., Case R.J., Stokes H.W. (2009). The integron/gene cassette system: An active player in bacterial adaptation. Methods Mol. Biol..

[15] Partridge S.R., Tsafnat G., Coiera E., Iredell J.R. (2009). Gene cassettes and cassette arrays in mobile resistance integrons. FEMS. Microbiol. Rev..

[16] Hall R.M., Collis C.M. (1995). Mobile gene cassettes and integrons: Capture and spread of genes by site-specific recombination. Mol. Microbiol..

[17] Chen T.L., Siu L.K., Wu R.C., Shaio M.F., Huang L.Y., Fung C.P., Lee C.M., Cho W.L. (2007). Comparison of one-tube multiplex PCR, automated ribotyping and intergenic spacer (ITS) sequencing for rapid identification of *Acinetobacter baumannii*. Clin. Microbiol. Infect..

[18] Zhang S.X., Rawte P., Brown S., Lo S., Siebert H., Pong-Porter S., Low D.E., Jamieson F.B. (2011). Evaluation of CLSI agar dilution method and Trek Sensititre broth microdilution panel for determining antimicrobial susceptibility of *Streptococcus pneumoniae*. J. Clin. Microbiol..

[19] Kristo I., Pitiriga V., Poulou A., Zarkotou O., Kimouli M., Pournaras S., Tsakris A. (2013). Susceptibility patterns to extended-spectrum cephalosporins among Enterobacteriaceae harbouring extended-spectrum β-lactamases using the updated Clinical and Laboratory Standards Institute interpretive criteria. Int. J. Antimicrob. Agents..

[20] Khorsi K., Messai Y., Hamidi M., Ammari H., Bakour R. (2015). High prevalence of multidrug-resistance in *Acinetobacter baumannii* and dissemination of carbapenemase-encoding genes *blaOXA-23-like*, *blaOXA-24-like* and *blaNDM-1* in Algiers hospitals. Asian. Pac. J. Trop. Med..

[21] Lee M.F., Peng C.F., Hsu H.J., Toh H.S. (2011). Use of inverse PCR for analysis of class 1 integrons carrying an unusual 3’ conserved segment structure. Antimicrob. Agents Chemother..

[22] Çıçek A.Ç., Düzgün A.Ö., Saral A., Kayman T., Çızmecı Z., Balcı P.Ö., Dal T., Fırat M., Tosun İ., Alıtntop Y.A. (2013). Detection of class 1 integron in *Acinetobacter baumannii* isolates collected from nine hospitals in Turkey. Asian. Pac. J. Trop. Biomed..

[23] Koeleman J.G., Stoof J., Van Der Bijl M.W., Vandenbroucke-Grauls C.M., Savelkoul P.H. (2001). Identification of epidemic strains of *Acinetobacter baumannii* by integrase gene PCR. J. Clin. Microbiol..

[24] Tenover F.C., Arbeit R.D., Goering R.V., Mickelsen P.A., Murray B.E., Persing D.H., Swaminathan B. (1995). Interpreting chromosomal DNA restriction patterns produced by pulsed-field gel electrophoresis: Criteria for bacterial strain typing. J. Clin. Microbiol..

[25] Huovinen P. (1999). Antibiotic usage and the incidence of resistance. Clin. Microbiol. Infect..

[26] Tenover F.C. (2006). Mechanisms of antimicrobial resistance in bacteria. Am. J. Infect. Control..

[27] Abbo A., Navon-Venezia S., Hammer-Muntz O., Krichali T., Siegman-Igra Y., Carmeli Y. (2005). Multidrug-resistant *Acinetobacter baumannii*. Emerg. Infect. Dis..

[28] Huddleston J.R. (2014). Horizontal gene transfer in the human gastrointestinal tract: Potential spread of antibiotic resistance genes. Infect. Drug. Resist..

[29] Leverstein-van Hall M.A., Box A.T., Blok H.E., Paauw A., Fluit A.C., Verhoef J. (2002). Evidence of extensive interspecies transfer of integron-mediated antimicrobial resistance genes among multidrug-resistant Enterobacteriaceae in a clinical setting. J. Infect. Dis..

[30] Krauland M.G., Marsh J.W., Paterson D.L., Harrison L.H. (2009). Integron-mediated multidrug resistance in a global collection of nontyphoidal *Salmonella enterica* isolates. Emerg. Infect. Dis..

[31] Hall R.M., Stokes H.W. (1993). Integrons: Novel DNA elements which capture genes by site-specific recombination. Genetica..

[32] Fluit A.C., Schmitz F.J. (2004). Resistance integrons and super-integrons. Clin. Microbiol. Infect..

[33] Taherikalani M., Maleki A., Sadeghifard N., Mohammadzadeh D., Soroush S., Asadollahi P., Asadollahi K., Emaneini M. (2011). Dissemination of class 1, 2 and 3 integrons among different multidrug resistant isolates of *Acinetobacter baumannii* in Tehran hospitals, Iran. Pol. J. Microbiol..

[34] Gu B., Tong M., Zhao W., Liu G., Ning M., Pan S., Zhao W. (2007). Prevalence and characterization of class I integrons among *Pseudomonas aeruginosa* and *Acinetobacter baumannii* isolates from patients in Nanjing, China. J. Clin. Microbiol..

[35] Ye Y.M., Li Z.D., Pan Y.P., Wei H. (2013). Association of integrons and inserted gene with antibiotics resistance of 100 strains of *Acinetobacter baumannii* isolates. Chin. J. Microecol..

[36] Fouad M., Attia A.S., Tawakkol W.M., Hashem A.M. (2013). Emergence of carbapenem-resistant Acinetobacter baumannii harboring the OXA-23 carbapenemase in intensive care units of Egyptian hospitals. Int. J. Infect. Dis..

[37] Lin M.F., Liou M.L., Tu C.C., Yeh H.W., Lan C.Y. (2013). Molecular epidemiology of integron-associated antimicrobial gene cassettes in the clinical isolates of *Acinetobacter baumannii* from northern Taiwan. Ann. Lab. Med..

[38] Gombac F., Riccio M.L., Rossolini G.M., Lagatolla C., Tonin E., Monti-Bragadin C., Lavenia A., Dolzani L. (2002). Molecular characterization of integrons in epidemiologically unrelated clinical isolates of *Acinetobacter baumannii* from Italian hospitals reveals a limited diversity of gene cassette arrays. Antimicrob. Agents Chemother..

[39] Nemec A., Dolzani L., Brisse S., van den Broek P., Dijkshoorn L. (2004). Diversity of aminoglycoside-resistance genes and their association with class 1 integrons among strains of pan-European *Acinetobacter baumannii* clones. J. Med. Microbiol..

[40] Zheng S.B., Ou J.M. (2010). Study on the integrons in *Acinetobacter* and the relationship with antibiotic resistance. Lab. Med. Clin..

